# Cell dynamics on energy landscape: Comparing attractor detection in Boolean network and diffusion‐based models under in silico gene perturbations

**DOI:** 10.1002/qub2.70056

**Published:** 2026-09-23

**Authors:** Lingyu Li, Liangjie Sun, Shumin Li, Wai‐Ki Ching, Zhi‐Ping Liu

**Affiliations:** ^1^ School of Biomedical Sciences The University of Hong Kong Hong Kong China; ^2^ Department of Mathematics The University of Hong Kong Hong Kong China; ^3^ Department of Biomedical Engineering School of Control Science and Engineering Shandong University Jinan China; ^4^ Institute for Life and Medical Sciences Kyoto University Kyoto Japan; ^5^ School of Artificial Intelligence Shenzhen University Shenzhen China

**Keywords:** Boolean network, cell‐state dynamics, energy landscape, gene perturbations, gene regulatory network, probabilistic diffusion equation

## Abstract

Steady‐state attractor detection differs fundamentally between discrete Boolean network (BN) and continuous diffusion‐based models because they operate on different state spaces and exhibit distinct dynamic behaviors. Here, we propose cell dynamics on energy landscape (CellLand) to systematically compare BN and diffusion‐based approaches for quantifying cellular dynamics on the energy landscape, with a particular focus on attractor identification under in silico gene perturbations. Although the energy landscape provides a unifying conceptual framework, BN‐based models face specific challenges, including state space explosion and pronounced sensitivity to network architecture, when assessing attractor stability and transition accessibility. Nevertheless, BN‐based analyses can remain reliable and interpretable when the underlying network topology is suitable, while being comparatively less sensitive to parameter estimation uncertainty and technical noise. Importantly, we show that attractor identification depends critically on network structure, and that no general theory currently exists for optimizing BN topology for dynamic cell‐state modeling. Our findings deepen the comparative understanding of BN and diffusion‐based frameworks and provide practical guidelines for selecting and designing models for robust simulation of complex gene‐regulatory systems under perturbations.

Computational models of cell‐state dynamics aim to represent, predict and ultimately steer cellular behavior in silico. By adopting a dynamical‐system perspective, such models describe how cellular states emerge, stabilize, and transition over time, enabling systematic analysis of robustness and responses to genetic or environmental perturbations. Within this framework, a steady state is a configuration in which all system variables remain constant over time in the absence of external perturbations [1]. Formally, it corresponds to a solution for which the time derivatives of all variables are zero, indicating that the system has reached dynamical equilibrium. Analyzing steady states is fundamental for understanding the long‐term behavior and stability of dynamical systems. In biological contexts, steady states (attractors) often correspond to meaningful phenotypes, such as distinct cell fates or physiological equilibria. Identifying and characterizing these attractors, that is, the steady states toward which the system naturally evolves, enables prediction of system responses to perturbations and elucidates possible transitions between different phenotypic and functional states.

Dynamical systems can be described using either discrete or continuous modeling frameworks. Boolean network (BN), first introduced by Kauffman in 1969 [2], is a commonly used discrete approach in which components assume binary values and are updated according to logical rules. BN models have a lot of practical applications in different areas such as systems biology, computer science and engineering, and are particularly effective for representing gene regulatory networks (GRNs) and cellular decision‐making processes. This success reflects that biological networks often depend on binary gene states rather than precise expression magnitudes [3]. Consistent with this, binarized expression suffices for spatial clustering and pattern discovery [4], validating Boolean abstractions of spatial transcriptomics. By contrast, continuous models employ real‐valued variables and differential equations to describe the evolution of the system. Among them, the probabilistic diffusion equation (i.e., diffusion‐based) framework is especially suitable for capturing stochastic dynamics and quantifying Waddington energy or pseudo‐energy landscapes in complex biological networks. Quantifying the phenotypic landscape of perturbed networks and identifying driver genes (or master regulators) that restore the landscape toward that of a reference (nominal) network are crucial for recovering realistic biological behavior and guiding therapeutic interventions.

Various modeling strategies have been used to study cellular phenotypic transitions by performing in silico gene perturbations on prescribed static GRNs, under the assumption that cellular identity is governed by complex regulation of gene expression [1]. BN‐based models are extensively used in this context; for example, Font‐Clos et al. applied a BN framework to investigate the landscape underlying epithelial‐mesenchymal transition (EMT) plasticity, revealing multiple steady states associated with distinct phenotypes [5]. Their study illustrates key challenges of steady‐state analysis in discrete systems, including the combinatorial explosion of possible network configurations and the sensitivity of attractor detection to network topology and update schemes. Similarly, continuous models, diffusion‐based models governed by Fokker‐Planck‐type equations, provide powerful tools for characterizing energy landscapes and the dynamics of complex biological networks [6]. For instance, Kang and Li proposed a dimension‐reduction strategy to study the energy landscape of a metabolism‐EMT network, enabling identification of intermediate and steady states [7].

More recently, machine‐learning‐based methods have substantially expanded the computational toolbox for landscape inference and dynamical reconstruction. Deep‐learning models such as EPR‐Net have enabled the direct inference of non‐equilibrium potential landscapes and steady‐state entropy production from high‐dimensional dynamics [8]. In parallel, Neural ODE and flow‐matching models have been used to reconstruct continuous cell‐state trajectories and latent dynamical laws from sparse single‐cell data [9]. Related data‐driven frameworks such as CellOracle infer GRNs from single‐cell multi‐omics data and perform in silico transcription‐factor perturbations to predict cell‐state transitions. Along a related geometric direction, GeoBridge models cellular dynamics on the transcriptional manifold and maps nonlinear trajectories to straight paths in a latent space, enabling biologically meaningful interpolation and reconstruction of unobserved intermediate states. Together, these studies show that cellular state transitions can be investigated from discrete, diffusion‐based, machine‐learning, and geometry‐informed perspectives, each offering complementary advantages for representing multistability, transition dynamics, and the organization of cellular‐state landscapes.

From the perspective of statistical physics, the evolution of a system can be viewed as motion on an energy landscape along dynamical trajectories. When thermal fluctuations are strong enough to overcome the barriers separating local minima, the system can transition between distinct basins of attraction. The smallest barrier between any two minima is associated with at least one intermediate, mixed configuration, which represents the most probable transition state visited as the system moves from one basin to the other. Motivated by this viewpoint, we analyzed three GRNs associated with hepatocellular carcinoma (HCC), melanoma, and a synthetic dataset generated by SERGIO. For each network, we constructed phenotypic/state landscapes by assigning pseudo‐energy values to stable attractor states embedded in a principal component analysis‐reduced space. We then systematically compared the cell‐state dynamics on energy landscapes obtained from a discrete BN description with those derived from a continuous diffusion‐based formulation, thereby elucidating both the differences between these frameworks and the distinct challenges they posed in identifying steady states, intermediate states, and transition pathways in complex gene regulatory systems under in silico gene perturbation.

In this study, we aim to identify master genes that drive cellular state transitions on a two‐dimensional (2D) pseudo‐energy landscape. Specifically, we seek mechanistic insight into the regulation of cell identity by evaluating how two dynamical‐system models (BN and diffusion‐based) capture phenotypic transitions induced by transcription factor (TF) perturbations. As an illustrative example, we first consider EMT and its reverse process, mesenchymal‐epithelial transition (MET), and then introduce our in silico perturbation strategy. Figure 1A schematically depicts EMT and MET. Epithelial (E) cells are typically non‐motile, whereas mesenchymal (M) cells exhibit migratory and invasive properties. Because EMT is reversible, cells may also occupy intermediate hybrid E/M states that co‐express epithelial and mesenchymal markers; on the pseudo‐energy landscape, these correspond to mixed states located between the E and M basins. Figure 1B outlines the in silico perturbation strategy used to test whether a given GRN can recapitulate TF perturbation‐induced changes in cell phenotype or fate following TF knockdown (KD) or overexpression (OE). The GRN is modeled as a directed, weighted TF‐target gene network. Perturbation of a TF is simulated by fixing its activity and propagating its influence on downstream target genes (TGs) along outgoing edges, with the magnitude of the effect determined by the corresponding edge weights. TF KD is implemented by setting the expression of the perturbed TF to 0 in the diffusion model and to −1 in the BN model, thereby defining an initial perturbed state vector for each cell. This state is then iteratively updated according to Supporting Information S1: Equation (2) for the diffusion model or Supporting Information S1: Equation (4) for the BN model until convergence. The resulting stable perturbed state yields a cell‐specific perturbation vector that encodes the predicted direction of fate change (Figure 1C, bottom). To enable direct application of the BN model to RNA‐seq data, we use a minimal binarization procedure (Figure 1C, top right). Reference E and M cell populations are first selected to define gene‐specific expression thresholds from their respective expression distributions. For each gene *i*, expression values above the threshold are assigned +1 (expressed), whereas values below the threshold are assigned −1 (not expressed). Using this unified framework, we analyzed three GRNs derived from HCC [5], melanoma [6], and SERGIO datasets, whose key structural properties are summarized in Table 1. In all three networks, each node assumes a binary state in the BN model (+1 for expressed and −1 for not expressed) and a continuous expression value in the diffusion model (nonzero for expressed and 0 for not expressed).

**FIGURE 1:**
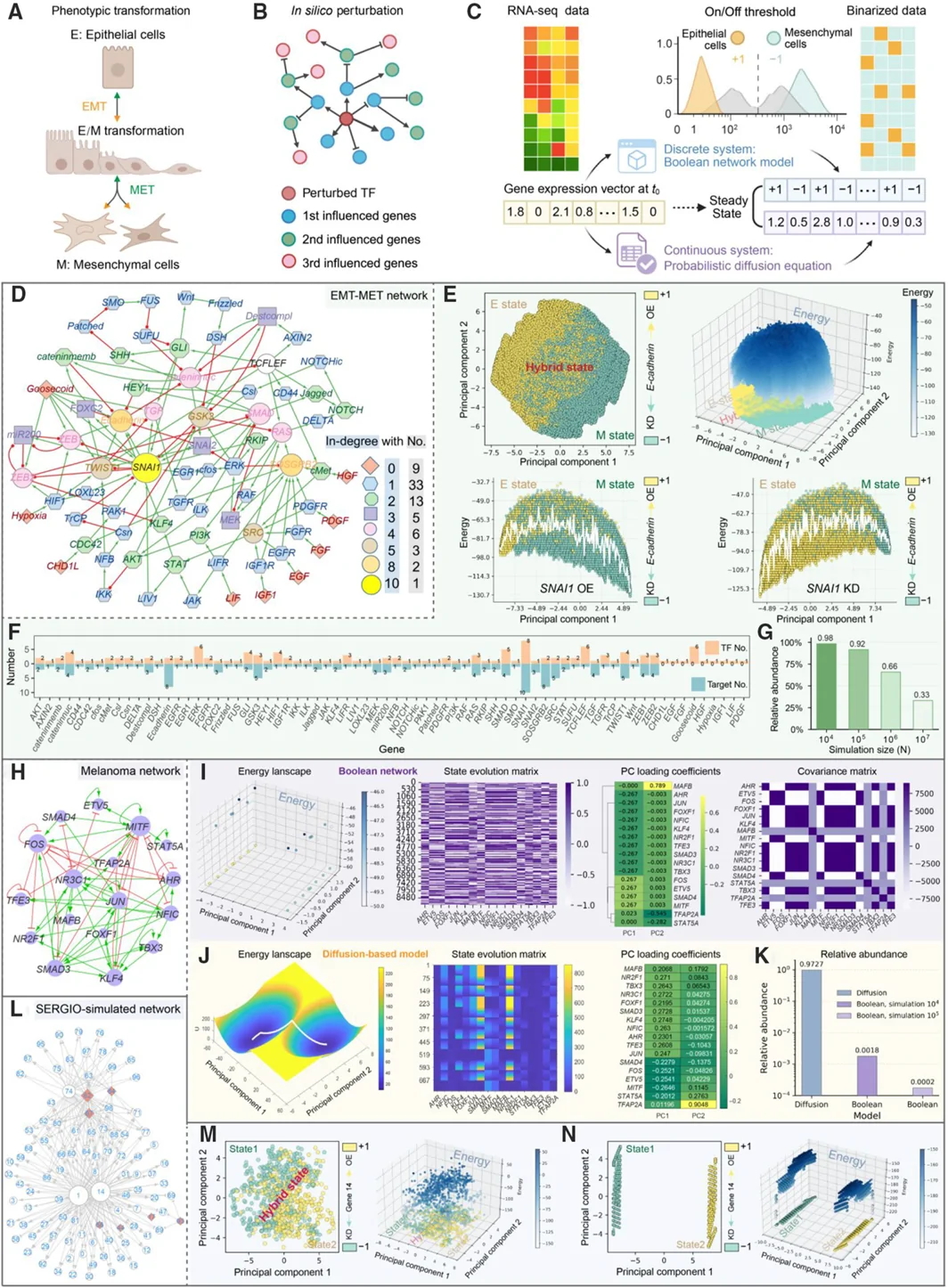
In silico perturbation framework and energy landscape analysis of GRNs to study cell phenotypic transitions via stable state attractors. (A) Schematic of phenotypic transitions between epithelial and mesenchymal cell states through EMT and MET. (B) In silico perturbation of a GRN, showing the perturbed TF and its first‐, second‐, and third‐order downstream influenced genes. (C) Overview of the in silico perturbation workflow for discrete and continuous dynamical systems, implemented using a BN model and probabilistic diffusion equations, respectively. In the discrete setting, RNA‐seq data are binarized using on/off thresholds defined from epithelial and mesenchymal reference states. In the continuous setting, the gene expression vector at *t*_0_ is evolved under probabilistic diffusion equations to reach a steady state. (D) EMT‐MET regulatory network in HCC. (E) State space and energy‐landscape analysis of the EMT‐MET network. Top left, PCA of 10^6^ steady states, showing the epithelial (E), mesenchymal (M) and intermediate hybrid regions; color denotes the E‐cadherin state. Top right, 3D reconstruction of the corresponding EMT energy landscape, in which higher‐energy states are concentrated in the transition region. Bottom, 1D PCA projections showing that *SNAI1* overexpression (OE) biases the landscape toward the M state, whereas *SNAI1* knockdown (KD) biases it toward the E state. (F) Number of TF regulators and downstream targets for each gene in the EMT‐MET network. (G) Relative abundance of steady states in the BN model across increasing numbers of initial conditions (N). Relative abundance is defined as the number of unique steady states divided by the number of initial conditions that converge to any steady state. (H) GRN in melanoma [6]. (I) BN‐model results for the melanoma network, including the energy landscape and the corresponding state‐evolution matrix, PC loading coefficients and covariance matrix. (J) Diffusion‐based model results for the melanoma network, including the energy landscape, and the corresponding state‐evolution matrix that describes the node expression trajectory matrix generated during diffusion‐based model simulation over time, and the heatmap of loading coefficients of each gene in the PC1 and PC2 directions after PCA. (K) Comparison of relative abundance between the diffusion model and the BN model; for the BN model, results from two simulation sizes are shown. (L) Denoised signed interaction network generated using SERGIO. (M–N) PCA projections of 10^4^ steady states and the corresponding energy landscapes obtained from Langevin dynamics (M) and from the BN model initialized from random states (N). BN, Boolean network; EMT, epithelial‐mesenchymal transition; GRNs, gene regulatory networks; HCC, hepatocellular carcinoma; MET, mesenchymal‐epithelial transition; PC, principal component; PCA, principal component analysis; TF, transcription factor.

**TABLE 1:** Summary of the interaction networks analyzed in this study.

	EMT^a^ ‐MET^b^ interaction network in HCC^c^	Gene regulatory network in melanoma	Signed interaction network in SERGIO
# nodes	72	17	100
# edges	139	64	258
Positive regulatory (→)	107	43	179
Negative regulatory (⊣)	35	21	79
# self‐loops	0	9	0
Avg. number of neighbors	3.694	5.294	5.160
Network diameter	13	4	2
Network radius	7	1	1
Characteristic path length	5.257	1.854	1.743
Clustering coefficient	0.400	0.291	0.012
Network density	0.027	0.202	0.026
Connected components	1	1	1
Multi‐edge node pairs	6	10	0

*Note*: Directed edges denoted by → indicate positive (activating) regulation from gene *i* to gene *j*, whereas edges denoted by ⊣ indicate negative (inhibitory) regulation.

^a^ EMT, epithelial‐mesenchymal transition.

^b^ MET, mesenchymal‐epithelial transition.

^c^ HCC, hepatocellular carcinoma. #, number.

Firstly, we studied the landscape of the EMT‐MET regulatory network in HCC. EMT operates in normal development and wound repair and is co‐opted during tumor dissemination. To determine how E–M plasticity is encoded by regulatory interactions, we applied the BN model in Supporting Information S1: Equation (4) to the EMT‐MET circuit in HCC (Figure 1D) to reconstruct the corresponding phenotypic landscape. A two‐dimensional principal component (PC) projection of 10^6^ simulations, each initiated from a random state and evolved to steady state, resolved the high‐dimensional state space into distinct attractor regions (Figure 1E). Steady states were classified as E, M, or hybrid states according to the expression state of E‐cadherin (*CDH1*). Mapping state abundance to the pseudo‐energy defined by Supporting Information S1: Equation (7) revealed a rugged landscape, in which E and M states occupied deeper basins, whereas hybrid E/M states were enriched in elevated intermediate regions (Figure 1E, top right; Supporting Information S1: Figure S1), consistent with their interpretation as metastable transition states. We next assessed how individual regulators reshape this landscape by fixing each node/gene *i* to +1 or −1, corresponding to OE or KD, respectively. OE of *SNAI1*, a canonical EMT inducer, shifted the 1D pseudo‐energy profile toward the M basin, whereas *SNAI1* KD biased the system toward the E state (Figure 1E, bottom). Thus, perturbation of a single TF can redirect attractor occupancy by reshaping the global landscape, indicating that the Boolean framework captures the essential regulatory constraints underlying EMT/MET plasticity [5]. The topological features of the EMT‐MET network further support this view (Figure 1F). The circuit comprises 72 nodes and 139 signed interactions, with an average degree of 3.694, a diameter of 13, and a radius of 7, indicating a sparse but globally connected architecture (Figure 1D; Table 1). Such an organization allows local perturbations to propagate through multi‐step regulatory cascades and generate system‐wide changes in attractor structure. Consistent with this, the in‐degree distribution shows that most nodes receive a moderate number of inputs (3–5), whereas only a few exhibit high in‐degree (8–10; Figure 1F). These highly connected nodes are therefore well positioned to integrate upstream signals and exert disproportionate control over cell‐state transitions, making them plausible master regulators of E–M plasticity. Finally, we examined the convergence of the estimated relative abundance of attractor states during the time‐evolution process (see Supporting Information S1: Supplementary Methods, Figure 1G). As the number of simulations increased from 10^4^ to 10^7^, the estimates became progressively more stable, supporting the robustness of the reconstructed landscape. Specifically, we examined the tendency of the relative abundance during the time‐evolution simulations (see Supporting Information S1: Supplementary Methods), defined as the fraction of sampled random initial conditions associated with distinct steady states. As the number of simulations increased from 10^4^ to 10^7^ (< 2^72^, approximately 4.72 × 10^21^), this abundance decreased but was higher than 25%, supporting the robustness of the reconstructed landscape (Supporting Information S1: Table S1).

Secondly, we studied the landscape for the melanoma GRN. Phenotypic heterogeneity in melanoma is closely associated with drug resistance, relapse and therapeutic failure. In addition to the canonical proliferative and invasive states, previous work identified a drug‐tolerant, hyperpigmented state marked by high expression of the lineage regulator *MITF* and its pigmentation targets (*PMEL*, *TYR* and *MLANA*). To dissect the regulatory logic and state‐transition trajectories underlying these phenotypes, a 17‐node GRN (Figure 1H) was analyzed and a diffusion‐based energy landscape (Supporting Information S1: Equation (3)) was reconstructed that accounts for four melanoma phenotypes [6]. Here, we analyzed the same network using both the BN model (Supporting Information S1: Equation (4)) and the diffusion‐based model defined in Supporting Information S1: Equation (3). As in the EMT‐MET analysis, we performed 10^4^ BN simulations from random initial conditions and classified the resulting states using *MITF* activity, which distinguishes proliferative from hyperpigmented programmes [6]. In contrast to the EMT‐MET network, the melanoma GRN has a distinct topology that strongly constrains Boolean dynamics. BN simulations therefore collapsed onto only 16 unique attractors despite extensive sampling of initial conditions (Supporting Information S1: Table S2). This degeneracy is evident in the state‐evolution and covariance matrices and produces a fragmented pseudo‐energy landscape composed of sparse, isolated states rather than smooth basins (Figure 1I). Among the 8998 simulations that converged to steady states, only 16 unique attractors were identified, and these could be grouped into five recurrent classes of node‐activity patterns (class 1: {6}; class 2: {13}; class 3: {15}; class 4: {1, 2, 7, 12}; class 5: {0, 3, 4, 5, 8, 9, 10, 11, 14, 16}), indicating that most genes remain locked in either the OE (+1) or KD (−1) state under the BN dynamics. Accordingly, the steady‐state matrix has rank 5, suggesting that the emergent attractor structure is dominated by network topology. By contrast, the diffusion model evolves continuous gene‐expression vectors under Supporting Information S1: Equation (3) and is not limited by this topological discretization. Under the selected parameterization (Supplementary Information Table S3–S6), the system relaxed to a dense ensemble of steady states, enabling reconstruction of a smooth energy landscape (Figure 1J). Consistent with this difference, the relative abundance of steady states, defined as the number of unique steady states divided by the number of initial conditions that converge to any steady state, was 0.9727 for the diffusion model, compared with 0.0018 and 0.0002 for BN simulations initiated from 10^4^ and 10^5^ states, respectively (Figure 1K; Supporting Information S1: Figure S2). Thus, for the melanoma GRN, the diffusion model more effectively captures the continuous organization of the state space and yields an interpretable energy landscape (Supporting Information S1: Figure S3), whereas the BN model collapses the dynamics into a small set of recurrent attractors imposed primarily by network topology.

Thirdly, we studied the landscape of a signed interaction network in a SERGIO‐simulated dataset. To further compare discrete and continuous dynamical formalisms for the identification of steady‐state attractors, we generated a synthetic single‐cell expression dataset using the diffusion‐based model (Supporting Information S1: Equation (3)) implemented in the SERGIO simulator, based on the ground‐truth signed GRN (Figure 1L). Specifically, we simulated a noise‐free steady‐state expression matrix simulated with sergio (number genes = 100, number bins = 3, number sc = 300, noise params = 1, decays = 0.8, sampling state = 15, noise type = ‘dpd’), resulting in three cell types (300 cells per type) across 100 genes (7 TFs and 93 TGs) connected by 258 regulatory edges. To evaluate the BN model (Supporting Information S1: Equation (4)), we binarized the continuous expression profiles as shown in Figure 1C. For each gene, the mean expression across the three cell types was used as the binarization threshold, resulting in a binary gene expression matrix. Gene 14, which has the highest degree in the network, was empirically selected as the classifier. In the diffusion‐derived steady‐state data, projection onto 2D PC space revealed two terminal states bridged by a substantial hybrid/intermediate population (Figure 1M, left). Consistently, the corresponding pseudo‐energy landscape, calculated using Supporting Information S1: Equation (7), placed these intermediate cells in a higher‐energy region between the two terminal basins (Figure 1M, right), indicative of a continuous transition structure in state space. By contrast, BN simulations performed on the same ground‐truth GRN (Figure 1L) from random initial conditions and iterated to steady state yielded only two sharply separated terminal states, with minimal occupancy of the intermediate region (Figure 1N, left). The corresponding 1D pseudo‐energy profile, calculated using Supporting Information S1: Equation (7), also failed to exhibit a meaningful topography (Figure 1N, right). Moreover, we also tried to increase the number of simulations to 10^5^ and 10^6^, but obtained very similar unsatisfactory results. These findings highlight the differences between continuous and discrete dynamical systems in steady‐state attractor detection. Although the BN model performs well for the EMT‐MET network with extensive simulations, the identification of distinct attractors remains highly sensitive to network structure.

Finally, we characterized cell‐state dynamics on the energy landscape with BN. In diffusion‐based systems, sensitivity primarily stems from model parameters, boundary conditions, and domain geometry, all of which strongly affect the existence and properties of stationary solutions. In fact, for BN models, steady‐state behavior depends not only on network topology, update rules (synchronous vs. asynchronous), and initial conditions, but also on the emergence of “frozen components”‐nodes locked in fixed states. Crucially, these frozen components smooth the underlying energy landscape, thereby driving the network toward orderly dynamics and facilitating adaptive evolution. As shown in Figure 1F, most nodes in the EMT‐MET network (Figure 1D) have moderate in‐degree values (from 3 to 5), whereas only a few exhibit high connectivity (from 8 to 10). These observations indicate that effective BN modeling requires a network of appropriate complexity; networks that are too simple or too complex may fail to capture diverse attractor states. Ideally, an optimized GRN should avoid redundancy, allowing for a rich set of diverse stable attractors and facilitating identification of minimal driver node sets that control long‐term BN dynamics. Under these conditions, BN models can efficiently capture biologically meaningful cell states or transitions, avoiding the extensive parameter tuning and computational demands typical of diffusion‐based approaches.

In this study, we utilized pseudo‐energy to quantify cell‐state dynamics under single‐node perturbations. Specifically, we measured the energy associated with long‐term network configurations following targeted perturbations, implemented by fixing the value of a node while initializing all others to maximal uncertainty. High pseudo‐energy indicates the potential for state transitions, whereas low values suggest restricted accessibility to other states. Using this framework, we successfully reproduced the established E/M hybrid state in the EMT‐MET network, as well as the *SNAI1* gene expression pattern associated with EMT plasticity (Figure 1E). Recent work by Parmer et al. [10] showed that the relative size of the optimal driver set in a network is determined solely by network degree, rather than overall network size or other structural features. Notably, nodes with zero in‐degree must be included in the driver set, whereas those with high degree centrality are more likely to belong to minimal driver sets. More broadly, network‐structural approaches suggest that certain subnetworks can provide useful indicators for distinguishing multiple steady states in chemical reaction networks and, in some cases, help determine all species concentrations uniquely. Collectively, these advances highlight the growing importance of integrating structural and dynamical perspectives to better understand and control complex biological networks.

In summary, we compared steady‐state detection and energy‐landscape analysis in BNs and probabilistic diffusion equations (diffusion‐based models). These two classes of models pose fundamentally different challenges, arising from their discrete versus continuous state spaces, the types of steady states they admit (point attractors and cycles vs. stationary distributions and metastable states), and the computational techniques required for their analysis. BNs are limited by combinatorial state‐space explosion and strong topology‐dependent attractor search, whereas diffusion‐based formulations require sophisticated analytical and numerical methods to resolve high‐dimensional stationary solutions. By systematically examining how BN and diffusion‐based frameworks differ in attractor structure, sensitivity to network topology, and responses to perturbations on energy landscapes, our study clarifies when each modeling paradigm is most appropriate for studying gene regulatory dynamics. This comparison highlights practical trade‐offs among computational cost, parameter sensitivity, and robustness to noise, and underscores the importance of aligning modeling choices with specific biological questions.

Recent methodological advances, including deep‐learning–based landscape construction (e.g., EPR‐Net) and neural ODE or flow‐matching approaches, provide powerful tools for learning non‐equilibrium potential landscapes and continuous‐time cell‐state dynamics from high‐dimensional data. These techniques can be applied to continuous stochastic models and, indirectly, to effective dynamics inferred from BN descriptions, thereby offering a bridge between mechanistic GRN‐based modeling and data‐driven landscape inference. Notably, our findings confirmed that the identification of influential attractors is highly dependent on the specific nature of network dynamics. As no unified theoretical framework yet exists to determine which types of networks, such as those defined by particular in‐degree or out‐degree constraints for certain nodes, are optimal for quantifying cell‐state dynamics on energy landscapes via BN models, our work helps delineate this design space and motivates future efforts to systematically optimize GRN structures and integrate discrete and continuous descriptions in multiscale models of cell‐state transitions.

## AUTHOR CONTRIBUTIONS

**Lingyu Li**: Conceptualization; investigation; methodology; data curation; validation; visualization; writing—original draft; writing—review and editing. **Liangjie Sun**: Methodology; writing—original draft; writing—review and editing. **Shumin Li**: Investigation; writing—original draft; writing–review and editing. **Wai‐Ki Ching**: Conceptualization; project administration; supervision; funding acquisition; writing—review and editing. **Zhi‐Ping Liu**: Conceptualization; project administration; supervision; funding acquisition; writing—review and editing.

## CONFLICT OF INTEREST STATEMENT

The authors declare no conflicts of interest.

## Supporting information

Supporting Information S1

## Data Availability

The EMT‐MET interaction network in hepatocellular carcinoma (HCC) was downloaded from the GitHub website (github.com/ComplexityBiosystems/bmodel/blob/master/bmodel/assets/topo_files/EMT_MET.topo). The gene regulatory network in the melanoma tumor was downloaded from the Github website (github.com/csbBSSE/Melanoma/blob/main/melanoma.topo). The signed interaction network in SERGIO was simulated using the SERGIO package (github.com/PayamDiba/SERGIO). All Python and MATLAB scripts, along with the input and output data used in this study, are available in the GitHub repository (github.com/zpliulab/CellLand). All data analyzed in this work are available through the figshare website (figshare.com/articles/dataset/CellLand_supplementary_data/32055507).
