# Supplementary material for: Cell dynamics on energy landscape: Comparing attractor detection in Boolean network and diffusion‐based models under in silico gene perturbations
Source: Quant Biol. 2026 Sep 23;14(4):e70056. doi: 10.1002/qub2.70056 (PMC13642054; doi:10.1002/qub2.70056)
Supplement: Supplementary file 1 [file QUB2-14-e70056-s001.pdf]

Supplemental Information for

"Cell dynamics on energy landscape: Comparing attractor detection in Boolean network and diffusion-based models under *in silico* gene perturbations"

Lingyu Li[1,2,3], Liangjie Sun[2,4], Shumin Li[1,5], Wai-Ki Ching[2,*], Zhi-Ping Liu[3,*]

[1]School of Biomedical Sciences, The University of Hong Kong, Hong Kong SAR, China
[2]Department of Mathematics, The University of Hong Kong, Hong Kong SAR, China
[3]Department of Biomedical Engineering, School of Control Science and Engineering, Shandong University, Jinan, 250061, China
[4]Institute for Life and Medical Sciences, Kyoto University, Kyoto, 606-8507, Japan
[5]School of Artificial Intelligence, Shenzhen University, Shenzhen, 518061, China

*Corresponding author: wching@hku.hk, zpliu@sdu.edu.cn

Contents

S1 Supplementary Methods **2**
S1.1 Dynamical System Models 2
S1.2 Challenges in Steady-State Detection 3
S1.3 Characterizing High-Dimensional Landscape 3
S1.4 Relative Abundance 4

S2 Supplementary Tables **4**
Table S1. Relative abundance of the EMT-MET network estimated from simulations of increasing size. 4
Table S2. Summary of steady-state statistics for the Melanoma network under diffusion and Boolean simulations. 4
Table S3. Description of diffusion-based model parameters. 4
Table S4. Parameters for node: *AHR* 4
Table S5. Parameters for node: *MITF* 5
Table S6. Initial values of 17 nodes for the melanoma network 5

S3 Supplementary Figures **6**
Figure S1. Energy landscape reveals potential wells, barriers, and qualitative cell-fate transition tendencies. 6
Figure S3. Gene regulatory network in melanoma and the corresponding energy landscape inferred from 10^5 Boolean network simulation. 6
Figure S3. Gene regulatory network in melanoma and the corresponding energy landscape inferred from the diffusion-based model. 7

S1 Supplementary Methods

S1.1 Dynamical System Models

Probabilistic Diffusion Equations Probabilistic diffusion equations, typically represented by partial differential equations such as the Fokker-Planck equation (FPE) [1], describe the time evolution of the probability density function (PDF) of a system's state. These equations take a macroscopic, ensemble-level perspective, providing information about how the probability of finding a system in a particular state evolves over space and time. They are deterministic in the equation form, as the randomness is encoded in the probability distribution rather than in the equations themselves. Analytical or numerical solutions to these diffusion-based models yield the full statistical distribution of the system at any given time.

For example, with $p(\mathbf{x}, \mathbf{t})$ representing the density function, Kang and Li [2] described the probabilistic diffusion equation:

$$\frac{\partial p(\mathbf{x},t)}{\partial t} = -\sum_i \frac{\partial}{\partial x_i}\left[f_i(\mathbf{x},t)p(\mathbf{x},t)\right] + d\sum_i\sum_j \frac{\partial^2}{\partial x_i \partial x_j} p(\mathbf{x},t), \tag{1}$$

to determine the time evolution of the dynamical system that described by the Langevin equation:

$$\dot{\mathbf{x}}(t) = \mathbf{f}(\mathbf{x},\mathbf{t}) + \mathbf{\Gamma}(\mathbf{t}), \tag{2}$$

where $\mathbf{\Gamma}(\mathbf{t})$ is N-dimensional independent Gaussian white noise, $\mathbf{x}(\mathbf{t})$ is a stochastic vector (e.g., gene expression level), whose component $x_i(t)$ represents the level of component i. Likewise, $\mathbf{f}(\mathbf{x},\mathbf{t})$ is a vector whose component $f_i(\mathbf{x},\mathbf{t})$ denotes the deriving force of the following systerm for $i = 1, 2, \cdots, N$:

$$f_i(\mathbf{x},t) = \frac{dx_i(t)}{dt} = \sum_{j=1}^{N} \frac{A_{ji} \times x_j^n(t)}{S_{ji}^n + x_j^n(t)} + \sum_{j=1}^{N} \frac{B_{ji} \times S_{ji}^n}{S_{ji}^n + x_j^n(t)} - k \times x_i(t), \tag{3}$$

where k is the basal degradation rate of $x_i(t)$, S_{ji} is the threshold of a sigmoidal function, n is the Hill coefficient that determines the steepness of the sigmoidal function, A_{ji} and B_{ji} measures the strength of the activation and inhibition of j on i ($A_{ji} = 0$ or $B_{ji} = 0$ means that there is no activation or inhibition from component j to component i).

Eq. (2) presents the general form of a continuous (stochastic) dynamical model that describes the time evolution of the PDF of system variables $\mathbf{x}$. Steady-state solution is crucial for understanding the long-term statistical behavior and equilibrium properties of this system modeled by Eq. (2). Given the steady-state probability distribution, the corresponding energy landscape can be quantified by solving the probability distribution from the associated FPEs derived from Eq. (2).

Boolean Networks (BNs) BNs are discrete dynamical systems widely used to model complex systems, especially in cell biology [3] and neuroscience [4]. In a BN, each node represents a component of the system (such as a gene or neuron) and is assigned a binary state, typically denoted as +1 (active /expressed) or −1 (inactive/not expressed) [5]. The state of each node at a given time step is determined by a set of logical update rules that depend on the states of its neighbors, and the update of the network can be performed either synchronously (i.e., with all nodes updated simultaneously) or asynchronously (i.e., with nodes updated individually in a specified order or randomly). For more details on analysis and control of BNs, see for instance, Tatsuya [6].

For example, Font-Clos et al. [7] reconstructed a network of N nodes ($N = 72$) to investigate EMT in HCC [8],

$$s_i(t+1) = \mathrm{sign}\Big(\sum_{\mathrm{j}} \mathrm{J_{ij} s_j(t)}\Big), \tag{4}$$

where s_i is the binary variable and represents if the gene or factor i is expressed/present ($s_i = +1$) or not ($s_i = -1$), J_{ij} is the nonsymmetric matrix that encodes the regulatory relations between nodes i and j and represents if j promote i ($J_{ij} = +1$) or j suppress i ($J_{ij} = -1$). Eq. (4) describes a network in which modes evolve asynchronously according to whether the total promoting input to a node exceeds its total inhibitory input, the node keeps its current state if $\sum_j J_{ij} s_j(t) = 0$.

BNs have been extensively applied to model GRNs, where the binary state represents gene expression (activated/inhibited) [9; 5]. The steady state in a BN is defined as a configuration such that the state of the system remains unchanged under the updating rules, known as a fixed point. More generally, the system may exhibit cyclic attractors, where a set of states is visited in a repeating sequence. Both fixed points and limit cycles are considered attractors of the BN and are crucial for understanding the long-term behavior of the modeled regulatory system.

S1.2 Challenges in Steady-State Detection

In the context of diffusion equations, a steady state is defined as a stationary distribution or equilibrium solution where the probability distribution no longer changes with time. It is challenging to detect steady-state by solving the FPE (derived from the diffusion model in Eq. (2)) directly.

(1) **Difficulty of analytical solutions:** Analytical solutions of Diffusion-based models are often intractable, so one must resort to numerical methods, which can be computationally expensive.

(2) **Cost of numerical approximation:** The computational cost associated with discretizing the domain and ensuring convergence of numerical schemes can be substantial, especially for high-dimensional systems.

To address this, for Eq. (2), Kang and Li proposed the Trajectory Moment Expansion (TME) method, which approximates the PDF $p(\mathbf{x})$ of a high-dimensional system as a weighted sum of M Gaussian distributions obtained by solving moment equations. Specifically,

$$p(\mathbf{x}) = \sum_{j=1}^{M} \phi^j p^j(\mathbf{x}), \tag{5}$$

where $p^j(\mathbf{x})$ and ϕ^j represent the density function and the weight of the j-th stable state, respectively.

For the BN model described in Eq. (4), a network state $\mathbf{s}$ is considered to be a steady state if all of its nodes remain unchanged upon updating. However, detecting steady states in Eq. (4) presents significant challenges:

(1) **Multiple attractors:** BN dynamics often exhibits both fixed-point and cyclic attractors, which complicates the identification and classification of steady states.

(2) **High sensitivity:** The attractor landscape and the accessibility of steady states can be strongly affected by initial conditions and by the choice of update scheme (synchronous vs. asynchronous).

In terms of Eq. (4), Font-Clos et al. [7] have demonstrated that limit cycles are never observed in this asynchronous updating scheme. Therefore, the phenotypic landscape associated with any given network can be constructed by initializing the network from various initial (gene expression) conditions and evolving the system until a steady state is reached, i.e., until each s_i does not change. In this manner, a large number of distinct steady states can be obtained.

S1.3 Characterizing High-Dimensional Landscape

For the random variable $\mathbf{x}$ in Eq. (5), its expectation and covariance can be computed as follows:

$$\mu = \sum_{j=1}^{M} \phi^j \mu^j, \quad \Sigma = \sum_{j=1}^{M} \phi^j \left(\Sigma^j + \mu^j (\mu^j)^T\right) - \mu\mu^T, \tag{6}$$

where M is the total number of stable steady states, and μ^j and Σ^j, corresponding to the j-th stable state, denote the expectation vector and the invertible covariance matrix of $\mathbf{x}^j$, respectively. Utilizing the concept of PCA, let $\mathbf{z} = \mathbf{w}\mathbf{x}$, where $\mathbf{w} = (\mathbf{w_1}, \mathbf{w_2})$ are the eigenvectors corresponding to the two largest eigenvalues of the covariance matrix of $\mathbf{x}^j$. The joint density function in this reduced-dimensional space can be expressed as $p_z(\mathbf{z}) = \sum_{j=1}^{M} \phi^j p_z^j(\mathbf{z})$, where p_z^j is a multivariate normal distribution. In this way, the energy landscape in two dimensions can be computed as $U = -\ln p_z(\mathbf{z})$.

While for BN model, let H be the pseudo-Hamiltonian that associated to one steady state $\mathbf{s}$ from Eq. (4), with the following definition:

$$H = -\sum_{i,j} J_{ij} s_i s_j, \tag{7}$$

Eq. (7) translates into the Hamiltonian of an asymmetric random bond Ising model [10; 11], it measures how much a given steady state respects the constraints imposed by J_{ij} and then accordingly assigns a value of energy. Remarkably, the fixed points of Eq. (7) are local minima when the interaction matrix is strictly symmetric (i.e., $J_{ij} = J_{ji}$). Font-Clos et al. [7] have proved that H is lower under repeated application of the evolution rule in Eq. (4). Therefore, Eq. (7) provides a measure of network state stability, where states with lower H are more stable than those with higher H. Analogously, H can later be projected onto a 2D landscape using PCA, where the x-axis and the y-axis are represented by PC_0 and PC_1, respectively. This indicates that the variable H in the BN model plays a role analogous to the energy variable U in the Diffusion-based model, with the advantage that H does not depend on the distribution of stable state data.

S1.4 Relative Abundance

For Boolean network model simulations, we estimate the relative abundance A of a network by calculating the fraction of all the random initial conditions [7] that show multi-steady-state (or multistability [3]).

$$A = \frac{\sum S_{\text{Uss}}}{S_{\text{Tol}}}, \tag{8}$$

where S_{Uss} represents the set that includes all unique/different steady states, the summation sign $\sum$ is used to count the total number of elements in S_{Uss}, S_{Tol} represents the total number of all random initial states for which the network is simulated [3].

In addition, if S_{Uss} represents the set includes a fixed steady states, and the summation sign $\sum$ is used to count the repeated occurrences of the fixed steady state, then each steady state $\boldsymbol{s}$ corresponds a value of $A(\boldsymbol{s})$ [7].

S2 Supplementary Tables

Table S1. Relative abundance of the EMT-MET network estimated from simulations of increasing size.

**Simulation**	**Total initial conditions**	**Initial conditions reaching steady states**	**Unique steady states**	**Relative abundance (A)**
Sim1	1.00E+03	878	864	0.864000
Sim2	1.00E+04	8720	7986	0.798600
Sim3	1.00E+05	87474	57654	0.576540
Sim4	1.00E+06	874481	290428	0.290428

Table S2. Summary of steady-state statistics for the Melanoma network under diffusion and Boolean simulations.

**Methods**	**Total initial conditions**	**Initial conditions reaching steady states**	**Unique steady states**	**Relative abundance (A)**
Diffusion	61×12	732	712	0.972678
Boolean sim1	1.00E+04	8998	16	0.001600
Boolean sim2	1.00E+05	90464	16	1.76E-04
Boolean sim3	1.00E+06	904537	16	1.76E-05

Table S3. Description of diffusion-based model parameters.

**Parameter**	**Full name**	**Description**
Prod	Production rate	Basal production rate of node
Deg	Degradation rate	Degradation rate constant
Trd	Threshold	Threshold for regulatory interaction
Num	Hill coefficient	Cooperativity of regulation
Act	Activation strength	Maximum fold change ($\lambda > 1$)
Inh	Inhibition strength	Maximum fold change ($\lambda < 1$)

Note: 61×12 indicates 61 trajectories $\times$ 12 initial conditions.

Table S4. Parameters for node: *AHR*

Node	Interaction	ParameterType	Value
AHR	AHR	Prod	30.911245
AHR	AHR	Deg	0.569513
AHR	AHR→AHR	Trd	3.870755
AHR	AHR→AHR	Num	6
AHR	AHR→AHR	Inh	0.015272
AHR	AHR→FOS	Trd	2.457006
AHR	AHR→FOS	Num	6
AHR	AHR→FOS	Inh	0.028788
AHR	AHR→KLF4	Trd	4.712927
AHR	AHR→KLF4	Num	6
AHR	AHR→KLF4	Act	7.932587
AHR	AHR→SMAD3	Trd	4.644236
AHR	AHR→SMAD3	Num	5
AHR	AHR→SMAD3	Act	16.351259
AHR	AHR→MITF	Trd	3.824863
AHR	AHR→MITF	Num	2
AHR	AHR→MITF	Inh	0.033896
AHR	AHR→NR3C1	Trd	1.938539
AHR	AHR→NR3C1	Num	4
AHR	AHR→NR3C1	Act	31.731145

Table S5. Parameters for node: *MITF*

Node	Interaction	ParameterType	Value
MITF	MITF	Prod	4453.815797
MITF	MITF	Deg	0.713843
MITF	MITF→MITF	Trd	1.881673
MITF	MITF→MITF	Num	6
MITF	MITF→MITF	Act	76.398161
MITF	MITF→NFIC	Trd	1.845243
MITF	MITF→NFIC	Num	1
MITF	MITF→NFIC	Inh	0.05213
MITF	MITF→FOS	Trd	0.395633
MITF	MITF→FOS	Num	1
MITF	MITF→FOS	Act	83.545537
MITF	MITF→KLF4	Trd	3.217281
MITF	MITF→KLF4	Num	2
MITF	MITF→KLF4	Inh	0.014588
MITF	MITF→JUN	Trd	0.2095
MITF	MITF→JUN	Num	2
MITF	MITF→JUN	Inh	0.013922
MITF	MITF→STAT5A	Trd	2.731615
MITF	MITF→STAT5A	Num	1
MITF	MITF→STAT5A	Act	66.272594
MITF	MITF→TFE3	Trd	1.29291
MITF	MITF→TFE3	Num	1
MITF	MITF→TFE3	Inh	0.018961
MITF	MITF→ETV5	Trd	0.918236
MITF	MITF→ETV5	Num	5
MITF	MITF→ETV5	Act	73.281334

Note: Apart from *AHR* and *MITF*, the parameters for the remaining 15 nodes are available at `https://github.com/zpliulab/CellLand/blob/main/Code/diffusion/Melanoma_Landscape/params/Tables.csv` (see the entry labeled 'Fig. 1' in the 'Figure' column).

Table S6. Initial values of 17 nodes for the melanoma network.

ID	AHR	NFIC	FOS	KLF4	FOXF1	JUN	SMAD3	MITF	SMAD4	MAFB	NR3C1	NR2F1	STAT5A	TBX3	TFE3	ETV5	TFAP2A
1	0.790613902	0.003814475	15.52291086	5.10E-08	6.431337144	0.007296133	1.28E-05	123.850825	274.7956216	0.032317489	0.012094924	0.001204537	97.08378969	4.96E-06	0.006484585	70.20606027	0.821290703
2	4.074383509	0.286272227	3.721511775	7.90E-05	6.432347741	0.111090226	0.004639753	0.055823931	274.79556	0.041438441	0.057396528	0.002569576	0.215014193	9.28E-05	1.185238918	0.130635896	0.051064216
3	6.345942447	20.64186161	0.188249579	79.76466708	25.49311569	630.0549937	176.1864299	1.32E-06	5.735259419	137.2475016	540.2820398	216.9314894	0.214764204	23.35668755	24.79612825	0.001521413	5.066212095
4	6.345935542	20.64168366	0.180552672	0.052757807	7.084203208	0.04757506	2.149057129	0.006690801	5.735259419	137.247112	359.5354179	11.62215001	0.21476421	0.000460239	2.415329473	0.001521416	5.062197098
5	0.46019599	0.047762899	2.637306104	8.22E-08	0.695164559	0.000109509	0.000273644	440.5565232	15.20915936	0.000381646	2.66E-05	3.23E-06	56.49234862	1.12E-06	0.033157265	64.1601485	8.115374113
6	0.460195913	0.047762899	2.637306191	8.22E-08	0.695164559	0.000109509	0.000273644	440.5565234	15.20915936	0.000381646	2.66E-05	3.23E-06	2.84359317	1.12E-06	0.033157261	64.1601485	8.115374113
7	8.079213803	85.4534742	0.002029432	1295.785601	45.78641794	102.936709	1129.263621	7.43E-06	1.608958185	0.028273519	33.13039745	23.95238891	0.086501357	35.91378604	14.52536646	0.010894967	3.463984489
8	4.017554063	1.8088146	0.092605953	0.000308069	0.695164559	0.003889628	0.044657551	0.399794338	15.20806997	0.000381646	0.000136275	0.001124183	0.437982534	2.46E-05	1.908642773	24.05935675	0.152954952
9	2.489549213	0.04505778	0.458237463	1.28E-05	0.644141969	1.06E-05	0.002365073	570.8159071	9.544446169	0.058318141	0.005642759	2.85E-05	97.54443194	4.06E-06	0.006290655	37.20177094	10.49162374
10	10.24753041	35.75727198	0.018737846	5.07E+00	62.51919903	0.060353196	7.96E+00	5.600010229	0.145259571	67.64255006	9.793014088	62.2848359	93.88662275	3.98E+01	0.205270017	0.23904525	578.9126466
11	10.26533017	92.84454216	7.94E-05	5.63E+01	62.54764203	0.411945458	75.62044494	0.005522958	0.145132853	81.99434051	75.70844633	76.89166866	0.021920693	6.46E+01	2.425435086	0.018624529	6.72270509
12	10.26533138	92.99244593	3.00E-05	1228.572525	62.54766281	34.69913098	741.8986957	1.23E-04	0.145132848	82.03141291	622.4652839	76.93216975	0.021920692	64.63245602	9.631199688	0.018565923	6.72270509
13	6.910112014	20.60528849	0.000176324	5.636370901	134.3884198	0.017433188	119.295341	2.40E-05	0.097918328	166.8559843	43.65015654	236.1583186	0.049106757	12.82309882	2.558010419	0.002433898	0.362321186
14	6.910111874	20.60528239	0.0001775	0.338209253	23.99321283	0.000220961	119.2953264	0.000682993	0.097918328	166.8559836	43.65015303	91.069696	0.049106757	0.001748254	2.413110708	0.002433898	0.385003321
15	1.002769038	0.3998777	0.545824959	0.000111375	23.78235318	3.13E-06	0.905173965	0.041950938	6.516543326	0.072659439	0.013961163	0.001053768	0.049106758	2.12E-06	0.415098158	0.076350269	0.022738591
16	0.076398573	0.399861737	3.639158508	1.81E-05	23.78235318	2.62E-06	0.058816823	0.042339865	6.516547013	0.050314673	1.52E-05	0.000561798	0.049106758	1.66E-06	0.004831798	0.07635364	0.022898134
17	0.022352267	0.040967305	7.236452469	2.88E-07	23.78235318	1.63E-07	0.001304491	4.073872889	6.516547013	0.050299632	5.87E-06	0.00014235	133.1022199	1.66E-06	0.000234667	17.64709871	0.335574884
18	4.87E-07	4.14E-06	3.084827594	4.63E-06	23.78235318	2.62E-06	0.004654585	0.042341203	6.516547013	0.05033268	2.64E-05	0.000685799	0.049106758	1.66E-06	0.004950661	0.076353649	0.022898683
19	9.66E-08	4.24E-07	7.242724048	2.25E-07	23.78235318	1.63E-07	0.001286979	4.073872874	6.516547013	0.05029963	5.87E-06	0.000142055	133.1022196	1.66E-06	0.000234667	17.64709871	0.335574884

S3 Supplementary Figures

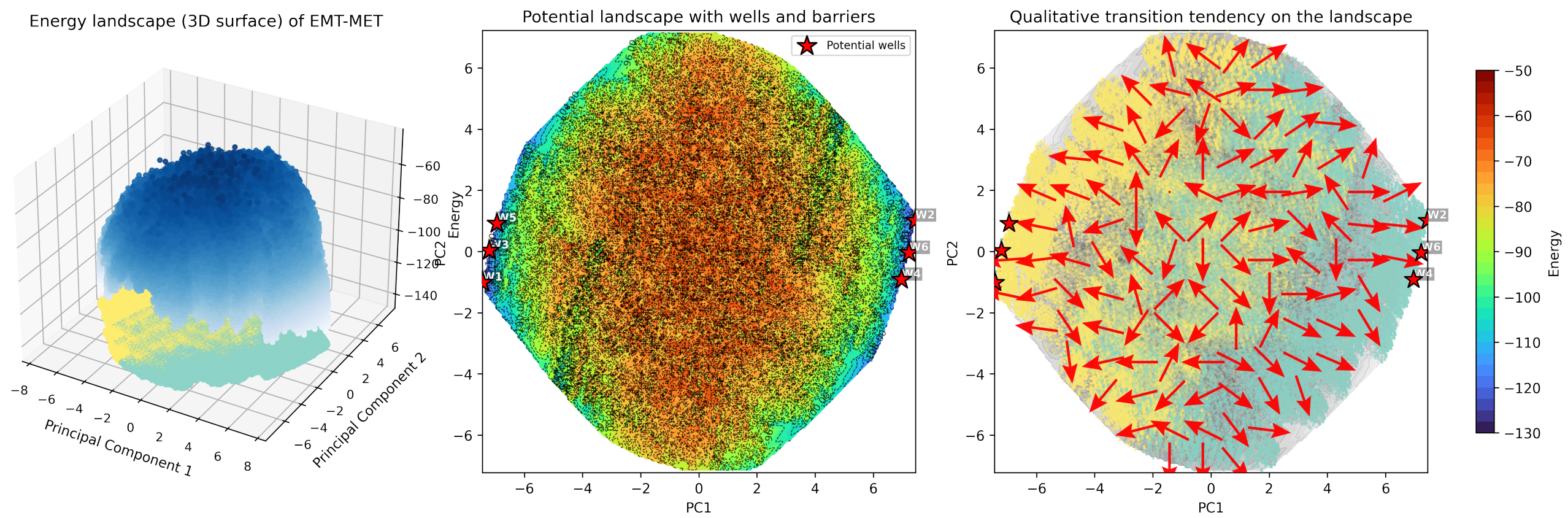

Figure S1. Energy landscape reveals potential wells, barriers, and qualitative cell-fate transition tendencies. (Left) The landscape is reconstructed in the space of the first two principal components (PC1 and PC2), where lower energy corresponds to more stable cell states. **(Middle)** A color-coded contour map (warm colors: higher energy; cool colors: lower energy) highlights basin-like wells and separating barriers; local low-energy attractors are marked by red stars. **(Right)** On a muted grayscale energy background, red arrows denote the inferred direction of state transitions from high- to low-energy regions, providing a qualitative view of putative cell-fate progression trajectories.

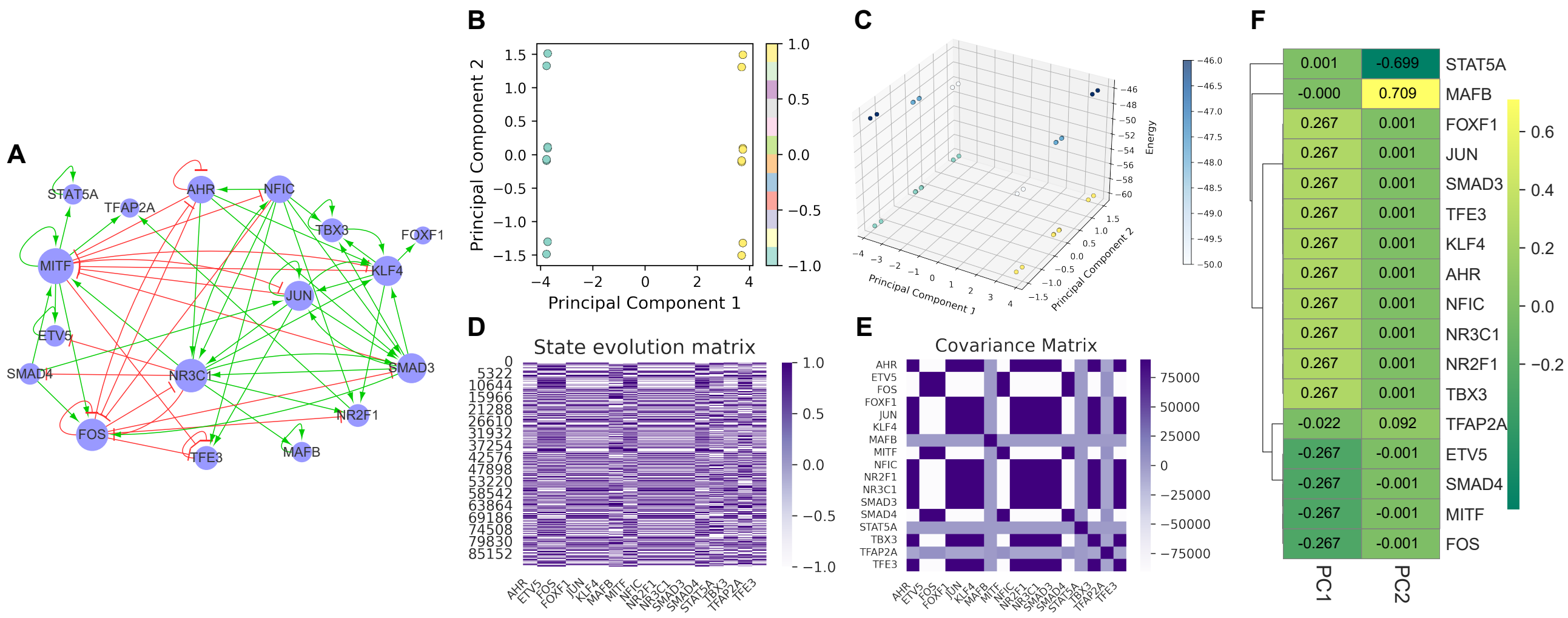

Figure S2. Gene regulatory network in melanoma and the corresponding energy landscape inferred from 10^5 Boolean network simulations. (A) Gene regulatory network of the melanoma system [12]. **(B)** Two-dimensional projection of the simulated steady states onto principal components 1 and 2. **(C)** Pseudo-energy landscape of the simulated steady states in the principal component space. **(D)** Corresponding state-evolution matrix. **(E)** Covariance matrix of the 17 network nodes. **(F)** Heatmap of the loading coefficients for principal components 1 and 2, i.e., heatmap of the contribution of variables on PC1 and PC2.

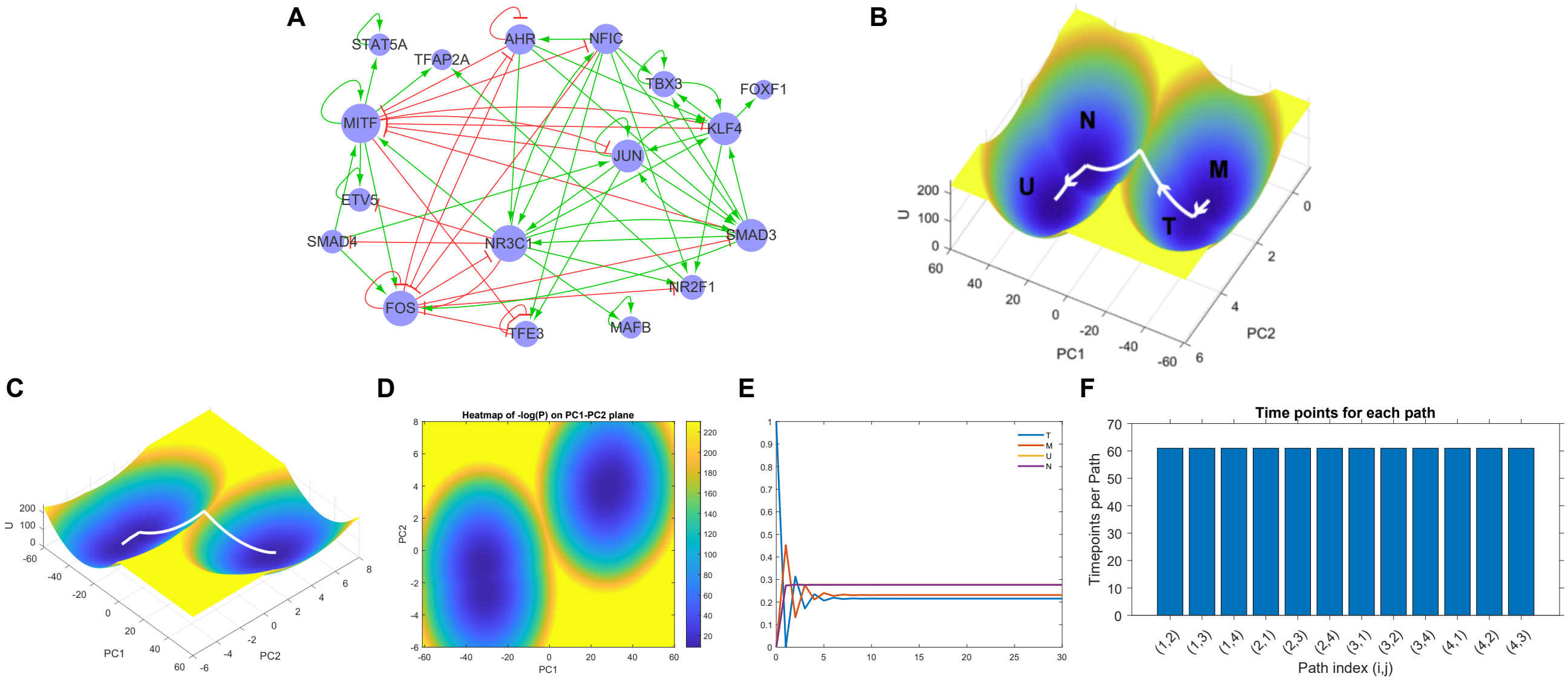

Figure S3. Gene regulatory network in melanoma and the corresponding energy landscape inferred from the diffusion-based model. **(A)** Gene regulatory network of the melanoma system [12]. **(B)** Energy landscape for the parameter set giving rise to four stable states/phenotypes, as reported in the original study [12]. **(C)** Reproduced energy landscape obtained using the original reproducible code. **(D)** Two-dimensional projection of the energy landscape shown as a heatmap on the PC1-PC2 plane, where the horizontal axis denotes PC1, the vertical axis denotes PC2, and the color scale represents the energy, $-\log(P)$. **(E)** Temporal evolution of the transition probabilities among the four phenotypic states during simulation. **(F)** Number of sampled time points for each trajectory when solving the diffusion-based model. Each trajectory is stored as $y_{\text{cell}_{i,j}} \in \mathbb{R}^{\#\ \text{nodes} \times T}$, where the number of sampled points is determined by the AMAM algorithm parameter *params.N*, corresponding to the number of points along the minimum action path.

Supplementary References

1. Risken, H. Fokker-planck equation 63–95 (1989).
2. Kang, X. & Li, C. A dimension reduction approach for energy landscape: identifying intermediate states in metabolism-EMT network. *Advanced Science* **8**, 2003133 (2021).
3. Rashid, M., Hari, K., Thampi, J., Santhosh, N. K. & Jolly, M. K. Network topology metrics explaining enrichment of hybrid epithelial/mesenchymal phenotypes in metastasis. *PLoS Computational Biology* **18**, e1010687 (2022).
4. Lynn, C. W. & Bassett, D. S. The physics of brain network structure, function and control. *Nature Reviews Physics* **1**, 318–332 (2019).
5. Li, L. *et al.* LogBTF: gene regulatory network inference using Boolean threshold network model from single-cell gene expression data. *Bioinformatics* **39**, btad256 (2023).
6. Akutsu, T. Algorithms for analysis, inference, and control of Boolean networks (2018).
7. Font-Clos, F., Zapperi, S. & La Porta, C. A. Topography of epithelial–mesenchymal plasticity. *Proceedings of the National Academy of Sciences* **115**, 5902–5907 (2018).
8. Steinway, S. N. *et al.* Network modeling of TGF-β signaling in hepatocellular carcinoma epithelial-to-mesenchymal transition reveals joint sonic hedgehog and Wnt pathway activation. *Cancer Research* **74**, 5963–5977 (2014).
9. Zhang, S.-Q., Ching, W.-K., Ng, M. K. & Akutsu, T. Simulation study in probabilistic Boolean network models for genetic regulatory networks. *International Journal of Data Mining and Bioinformatics* **1**, 217–240 (2007).
10. Binder, K., Heermann, D. W. & Binder, K. Monte Carlo simulation in statistical physics **8** (1992).
11. McCoy, B. M. & Wu, T. T. The two-dimensional Ising model (2014).
12. Pillai, M. & Jolly, M. K. Systems-level network modeling deciphers the master regulators of phenotypic plasticity and heterogeneity in melanoma. *iScience* **24**, 103111 (2021).
